# Chemical and Microscopical Analysis of the Urine, in Health and in Disease

**Published:** 1877-02

**Authors:** 


Chemical and Microscopical Analysis of the Urine, in Health and in Disease. Designed for physicians and students. By Geo. B. Fowler, M.D., Examiner in Physiology, College of Physicians and Surgeons, New York; Visiting Surgeon to the New York Dispensary; Fellow of the New York Academy of Medicine; Member of the New York County

Medical Society, etc. Second edition, revised and enlarged, with eighteen illustrations. G. P. Putnam's Sons, publishers: New York. 1876.

This volume of 97 pages is a hand-book of analyses of the urine; and one, too, qualified to be of considerable benefit to physicians as well as students. It deals in the physical and chemical characters of the urine in a state of health and disease, and enters critically into the analyses of the secretion in what ever condition it may be.

Part 1 deals, first, in the characters; and second, in the effects of reagents in normal urine. Part 2 treats of the characters of abnormal urine. Part 3 classifies the urinary deposits. Part 4 gives the accidental ingredients which do not form deposits. Part 5 consists of a very important chapter on quantitative analysis; and Part 6 is devoted to the constituents, formation, and character of calculi and gravel.

It is a book that will fill the wants of many, and no doubt will meet with a good reception.

